# Electrospun Polymers in Cartilage Engineering—State of Play

**DOI:** 10.3389/fbioe.2020.00077

**Published:** 2020-02-18

**Authors:** Elif Nur Yilmaz, Dimitrios I. Zeugolis

**Affiliations:** ^1^Regenerative, Modular & Developmental Engineering Laboratory, National University of Ireland Galway, Galway, Ireland; ^2^Science Foundation Ireland, Centre for Research in Medical Devices, National University of Ireland Galway, Galway, Ireland

**Keywords:** electrospinning, fibrous scaffolds, cartilage engineering, functionalised scaffolds, *in vivo* models

## Abstract

Articular cartilage defects remain a clinical challenge. Articular cartilage defects progress to osteoarthritis, which negatively (e.g., remarkable pain, decreased mobility, distress) affects millions of people worldwide and is associated with excessive healthcare costs. Surgical procedures and cell-based therapies have failed to deliver a functional therapy. To this end, tissue engineering therapies provide a promise to deliver a functional cartilage substitute. Among the various scaffold fabrication technologies available, electrospinning is continuously gaining pace, as it can produce nano- to micro- fibrous scaffolds that imitate architectural features of native extracellular matrix supramolecular assemblies and can deliver variable cell populations and bioactive molecules. Herein, we comprehensively review advancements and shortfalls of various electrospun scaffolds in cartilage engineering.

## Introduction

Adult articular cartilage is a relatively thin (2–4 mm), aneural, avascular, and alymphatic tissue that acts as cushion against physiological loads at joints. Once injured, it loses much of its carrying capacity, causing a susceptible environment for wearing and tearing between the joints (Correa and Lietman, 2017; Zhang et al., 2019). It has been reported that 60–66% of routine knee arthroscopies caused by articular cartilage defects. The breakdown molecules following injury cause an inflammation in the joints. This inflammation increases the level of synovial cytokines, alters the resident cell phenotypes and induces matrix-degrading enzymes. Thus, it causes a more conducive environment for tissue degradation, which finally ends up with osteoarthritis (OA) (Homandberg et al., 1993; Homandberg and Hui, 1996; Cecil et al., 2005; Kurz et al., 2005; Goldring et al., 2011; Camp et al., 2014). Conjecturally, up to 240 million people around the world suffer from OA. The observed symptoms (e.g., pain, stiffness, joint instability, and pain-related psychological distress) start approximately at the age of 55 and have devastating consequences in the quality of life of the patients (Hunter et al., 2008; Van Spil et al., 2019). In 2013, OA was the second most expensive health condition treated at US hospitals with $16.5 billion expenditure (Torio and Moore, 2013). Women have a higher age-related prevalence of arthritis than men, 10% men and 13% in women suffer from aged-related OA (aged 60 years or older) (Zhang and Jordan, 2010). This prevalence is projected to increase due to increasing aging population and obesity (Sun et al., 2015).

There are numerous treatments for articular cartilage defects, including extensive surgical interventions (e.g., osteotomy, distraction of joints), therapeutic interventions without active biologics (e.g., lavage, arthroscopy, debridement, shaving, laser chondroplasty, abrasion chondroplasty, pridie drilling, microfracture, and spongialization), therapeutic interventions with active biologics (e.g., perichondrial/periosteal grafts, osteochondral transplantation, allogenic osteochondral, and chondral grafting) and tissue engineering (a still elusive combination of scaffolds, cells, biologics). Cartilage engineering constitute the ultimate frontier, as all other interventions are nothing more than relieving the pain or delaying tissue degradation (Hunziker, 2002; Musumeci et al., 2014). Various scaffold fabrication technologies have been assessed over the years for cartilage engineering with variable degree of efficiency (Cheng et al., 2019; Li et al., 2019). Among them, electrospinning has emerged as a promising technique, due to its high versatility (e.g., ability to produce functionalised nanofibrous scaffolds with a variety of orientations, sizes, and mechanical properties) (Garg and Bowlin, 2011; Casanellas et al., 2018; Casanova et al., 2018; Li et al., 2018; Liu et al., 2018). Herein, we briefly describe the cellular and extracellular composition and architecture of cartilage, along with key modulators of chondrogenesis, and we comprehensively review advancements and shortfalls of electrospun scaffolds in cartilage engineering.

## Cartilage

### Cartilage Cellular Composition and Key Signaling Molecules in Chondrogenesis

Cartilage is a hypocellular tissue, with only 4% of its wet weight consisting of a highly differentiated cell population, called chondrocytes (Matzat et al., 2013). The morphology of chondrocytes varies in shape in each zone (see section Cartilage extracellular matrix composition and architecture). Chondrocytes together with the pericellular matrix (a basket-like network of fine fibrils of elaborate structure composed of laminin, fibronectin, biglycan, decorin, fibromodulin, matrilin 3, and cartilage oligo matrix protein) and the capsule (composed of collagen type VI, collagen type IX, and proteoglycans) surrounding the pericellular matrix form the chondron, which reduces the mechanical, osmotic and physicochemical changes induced by dynamic loading, maintain tissue homoeostasis and contribute to tissue regeneration (Muir, 1995; Alexopoulos et al., 2003; Youn et al., 2006; Vonk et al., 2014; Wilusz et al., 2014; Decker et al., 2015; Li and Xu, 2015). Alterations in the composition of the pericellular matrix is associated with OA (Wadhwa et al., 2005a,b; Hu et al., 2006; van der Weyden et al., 2006; Alexopoulos et al., 2009). Chondrocytes are responsible for synthesis of the articular cartilage extracellular matrix (ECM) (Bhosale and Richardson, 2008; Demoor et al., 2014) and its remodeling through secreted enzymes (e.g., matrix metalloproteinases, hyaluronidases, aggrecanases) (Buttle et al., 1997; Shlopov et al., 1997; Flannery et al., 1998; Demoor et al., 2014).

Chondrocytes are originated from mesenchymal stem cells (MSCs), found in the bone marrow of mature individuals. Condensation of MSCs and chondroprogenitor cell differentiation initiate cartilage formation. Expression of collagen type I and type II results in the onset of chondrogenesis (Archer and Francis-West, 2003; Demoor et al., 2014). Pre-chondrocytes start expressing cartilage-specific transcription factors (e.g., Sox9, Sox5, Sox6) and then they become mature chondrocytes by producing an ECM that has a great amount of proteoglycans (e.g., aggrecan) and collagens (e.g., collagen types II, IX, and XI) (Bi et al., 1999; Ikeda et al., 2004; Demoor et al., 2014). As the chondrocytes proliferate, they express collagen type VI and matrilin 1 under the control of the parathyroid hormone-related peptide/Indian hedgehog. Indian hedgehog is a secreted factor in hypertrophic chondrocytes, which is regulated by the activation of the cyclins. The cyclins regulate chondrocyte proliferation via formation of complexes with cyclin-dependent kinases. By secreting the cartilaginous matrix, MSCs differentiate to chondrocytes and they continue to divide during chondrogenesis. At the final step of their development, they become hypertrophic and secrete calcification proteins in the calcified zone (Temenoff and Mikos, 2000; Zelzer et al., 2001; Goldring, 2012).

**Figure 1:**
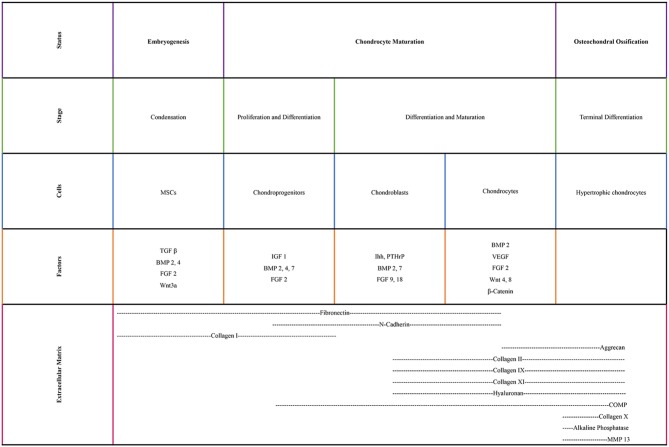
Biological factors and extracellular molecules involved in chondrogenesis.

Various transcription factors are crucial in chondrogenesis (Figure 1). Sox9, which is a master chondrogenic transcription factor during the chondrogenic differentiation, upregulates the transcriptional activity of collagen type II gene through interacting with the first intron-specific enhancer. Sox9 is crucial for articular cartilage formation and the hypertrophic maturation of chondrocytes. In the absence of Sox9, Sox5, and Sox6 induce the transcriptional activity of collagen type II gene, albeit slightly. These three members of the Sox family also regulate the gene expression of collagen type IX, collagen type XI and aggrecan (Lefebvre and Smits, 2005; Wuelling and Vortkamp, 2011; Demoor et al., 2014). Runx2 and Runx3 are expressed in pre-hypertrophic and hypertrophic chondrocytes. Deletion of Runx2 and Runx3 delays chondrocyte maturation. Hypertrophic chondrocytes cannot be formed when lacking these two transcription factors (Yoshida et al., 2004). c-Maf is a basic leucine zipper transcriptional activator and allows hypertrophic and terminal chondrocytes to terminally differentiate (MacLean et al., 2003; Lefebvre and Smits, 2005).

Growth factors also play key roles in chondrogenesis. Insulin-like growth factor1 induces collagen type II expression through increased binding activity of Sox trio (Seifarth et al., 2009; Renard et al., 2012; Legendre et al., 2013; Demoor et al., 2014). Transforming growth factor β1 initiates the condensation of MSCs to chondrocytes for the onset of chondrogenesis, increases the collagen type II gene expression levels during the early stage of chondrogenesis and inhibits the terminal differentiation of chondrocytes via increasing the expression of parathyroid hormone-related peptide (Li et al., 2005a; Demoor et al., 2014). Bone morphogenic protein 2 plays a pivotal role in the expression of the mature form of collagen type II (Rosen et al., 1994; Gouttenoire et al., 2010; Demoor et al., 2014).

WNT signaling is a well-studied pathway for differentiation and hypertrophy (Ripmeester et al., 2018). WNTs establish a large family of cysteine-rich morphogens that have an essential role in cartilage, bone and joint development. *In vivo* mice studies indicated that WNT signaling extended cell survival and inhibited the differentiation of chondrocytes toward hypertrophy (Zhu et al., 2008, 2009). WNT5a and WNT5b are important during differentiation of MSCs to chondrocytes (Church et al., 2002), chondrocyte proliferation and cartilage homeostasis (Sharma et al., 2013). However, overexpression of WNTs has been reported to lead to OA-like diseases (Lodewyckx and Lories, 2009).

The surface zone of articular cartilage contains a subpopulation called cartilage progenitor cells. These flat cells are responsible for the appositional growth of the cartilage tissue and express high level of stem cell surface marker (Hiraoka et al., 2006) and exhibit a significant degree of plasticity, in terms of differentiation toward chondrogenic, osteogenic, and adipogenic pathways (Morrison et al., 1997; Dowthwaite et al., 2004). Upon injury to a healthy cartilage, they migrate and emerged to the injury site. During OA progression, changes in the distribution of cartilage progenitors suggests that these cells may be responsible for communication between articular cartilage and subchondral bone (Jiang and Tuan, 2015).

### Cartilage Extracellular Matrix Composition and Architecture

Cartilage is mainly comprised of collagens (types II, VI, IX, X, XI); collagen type II is the predominant collagen that forms the 90–95% of the fibril network of the matrix and 60–85% of the dry weight of cartilage (Buckwalter and Mankin, 1997; Mow et al., 1999; Poole et al., 2001; Pearle et al., 2005; Lim et al., 2014). Bound carbohydrate groups found in collagen type II allow to interact with water more than other types of collagen. Together with collagen type II, types IX and XI form a macro-fibrillar structure/fiber network, which provides tensile strength. Collagen type IX is cross-linked to the surface of the macro-fibrils, whereas collagen type XI located within and on the surface of the macro-fibrils. Collagen type VI forms microfibrils in pericellular sites. Collagen type X is only synthesized by hypertrophic chondrocytes, which takes place in calcified cartilage (Cohen et al., 1998; Temenoff and Mikos, 2000; Poole et al., 2001). Proteoglycans consist of a protein core and one or more glycosaminoglycan chains. Hyaluronic acid, chondroitin sulfate, keratan sulfate, dermatan sulfate, and heparan sulfate are some of the glycosaminoglycans found in articular cartilage. The predominant proteoglycan is the large chondroitin sulfate proteoglycan 1, called aggrecan, which forms a strong, porous-permeable, fiber-reinforced material together with collagen fibrils. Aggrecan, as the name implies, forms an aggregate structure that does not allow proteoglycans to diffuse out of the matrix throughout joint loading, thus plays an important role during compressive loading. Decorin, biglycan, and fibromodulin are present in minor quantities and do not significantly affect the physical properties of the tissue, unlike aggrecan (Buckwalter and Mankin, 1997; Cohen et al., 1998).

**Figure 2:**
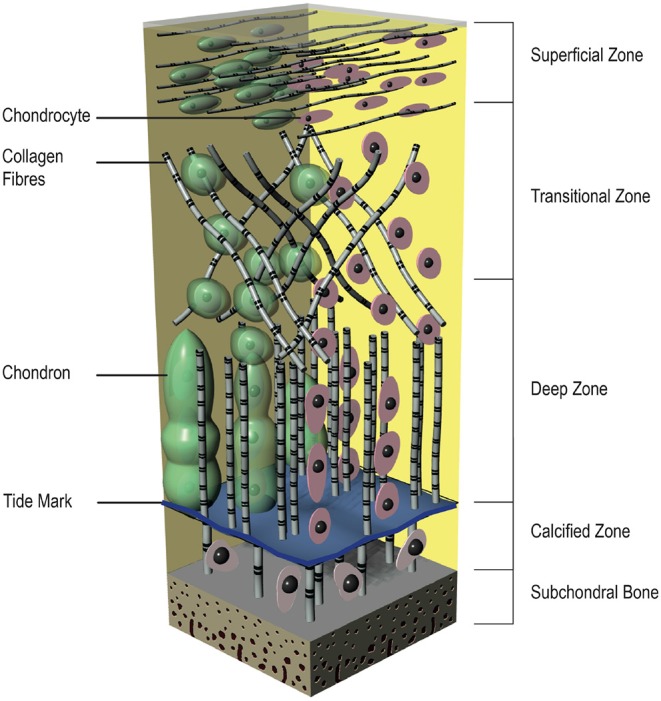
A simplified graphical illustration of articular cartilage.

From top to bottom the articular cartilage can be divided into four distinct layers with different compositions, cell morphologies, and physiological characteristics (Figure 2). The superficial zone is the thinnest zone, constitutes 10–20% of the total cartilage volume and is responsible for tensile properties of the tissue. It includes a high density of ellipsoid chondrocytes (24,000 cell/mm^3^) with a parallel orientation to the surface. These ellipsoid chondrocytes synthesize high concentration of collagens [mainly type II and type IX collagen fibers with small diameter (20 nm) and parallel arrangement to the surface] and low concentration of proteoglycans; for this reason, this zone has the highest concentration of water. As a result of its construction, this zone protects deeper zones from shear, tensile, and compressive forces. Below the superficial zone, the transitional zone represents 40–60% of the total cartilage volume and has a lower cell density (10,300 cells/mm^3^). This middle zone shows more typical morphologic features of a hyaline cartilage, with more spherical cells, higher fiber diameter and higher aggrecan content (Temenoff and Mikos, 2000; Poole et al., 2001; Bhosale and Richardson, 2008; Sohier et al., 2008; Nazempour and Van Wie, 2016). Situated between the transitional zone and the calcified cartilage is the deep zone, which represents almost 30% of the total cartilage volume. It provides a great strength against compressive forces and contains the lowest cell density among all of the zones (7,700 cells/mm^3^). The cells in this zone are large and spherical and organized perpendicularly to the joint surface. Although the lowest cell density, the proteoglycan content and the fiber diameter (120 nm) are maximal in this zone. Between the deep zone and the subchondral bone, the calcified zone is located and constitutes an excellent interface that integrates with less resilient subchondral bone. There is a visible border between the deep and calcified zone, called tidemark. The calcified zone has a small volume of ellipsoid cells with an abundant calcified ECM, shows a very low metabolic activity. The chondrocytes in this zone exhibit a hypertrophic phenotype and, uniquely, they express collagen type X that can calcify surrounding ECM (Sohier et al., 2008; Sophia Fox et al., 2009).

## Electrospinning

### The History

**Figure 3:**
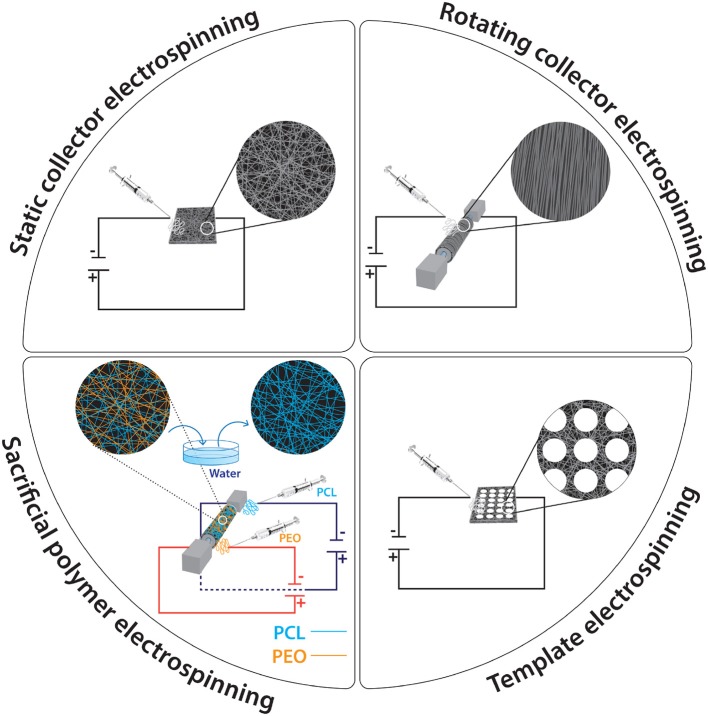
Widely used electrospinning setups.

Electrospinning is a highly versatile technique that produces ultrafine fibers with a diameter in the nano- to micro- meter range by using electrostatic fields. It has become popular in a wide range of biomedical and industrial applications, as it can produce fibrous mats with controlled orientations, sizes, porosity, mechanical properties, and with high surface area to volume ratio (Figure 3). In 1882, Lord Rayleigh first described electrospray, which inspired the idea of the electrospinning process. He investigated “The Rayleigh instability”; a highly charged droplet is unstable and would break down into smaller droplets when passes through a voltage gradient. After his initial work, the electrospraying of aqueous solutions achieved by the workmanship of Zeleny; his work made possible the current state of electrospinning. It is considered a direct extension of electrospraying, considering that continuous fibers are produced in electrospinning, whereas small droplets are produced in electrospraying. In 1934, Formhals achieved a feasible method to get fine fibers from a cellulose acetate solution and took out a variety of U.S. patents on this technology. In 1966, Simons observed that the use of more viscous solutions resulted in longer fibers. Later, Baumgarten discovered that the diameter of acrylic fibers could be controlled by the feed rate of the infusion pump. Despite these advances and patents in the field of electrospinning until the 1990s, there was no commercial interest in this technique. From the beginning of 1990's, as nanotechnology became a popular research area, the interest of electrospinning has increased (Li et al., 2007; Molnár and Vas, 2012; Braghirolli et al., 2014).

### The Setup

The electrospinning process is a simple, efficient spinning method that produces nanoscale to microscale fibers from polymer solutions or melts using electrostatic forces. It is a relatively easy to setup process, as it requires a syringe (polymer solution reservoir) with a small diameter needle (to charge the polymer solution), a flow control pump for reproducibility, a high voltage supply to produce a charged polymer jet and a collector. If the syringe is not set horizontally, the polymer flow can be driven by gravity. The voltage supply usually ranges from 10 to 50 kV (subject to solution viscosity, solvent volatility, etc.). The collector is usually a stationary plate, although advances in engineering have allowed the use of a rotating cylinder for the production of anisotropic fibers.

### The Process

Electrospinning begins when the polymer solution emerges from the spinneret by the electrostatic forces. While it is extruded from the syringe, it forms a semi-spherical droplet at the end of the needle and due to the induction of charges on the polymer droplet, it causes instability within the polymer solution. When the reciprocal repulsion of the charges is enough to overcome the surface tension, a conical shape cone, known as Taylor cone, is formed via the elongation of polymer droplet. A liquid jet is the formed that flows through the direction of electric field. During this journey from the spinneret to the collector, the solvent in the liquid jet evaporates, increasing the surface charge on the jet. This increasing surface charge causes instability in the polymer jet and the polymer jet divides geometrically to compensate for the instability. First, it divides into two jets and, as the process continues, it divides into more and more jets. The action of the spinning force, which is caused by the electrostatic force on the continuously splitting polymer droplets, produces the non-woven nanofibers, which are deposited on the collector (Li and Tuan, 2009; Liu et al., 2012; Haider et al., 2015). Despite the electrospinning process seems quite simple, a number of process parameters should be adjusted in order to get desired morphology of nanofibers without droplets or beads (Pillay et al., 2013). These parameters can be divided 3 major group: solution parameters; process parameters; and ambient parameters.

### Solution Parameters

All solution parameters (e.g., concentration, molecular weight, viscosity, surface tension, conductivity) are related to each other and affect the architectural features of the produced mat. The concentration of the solution is crucial for fiber formation to occur. Thus, an optimized solution concentration should be designated for each polymer. When the concentration is very low, the electrospinning process does not occur, instead of this, electrospraying is achieved via obtaining polymeric nano/micro particles, because of the low viscosity and high surface tensions of the solution. As the concentration goes a little higher, a mixture of beads and fibers occurs. A further increase in concentration changes the bead morphology from spherical to spindle-like. When the concentration reaches a suitable level, smooth fibers are obtained. Above this level, an increase in concentration results in an increase in fiber diameter. If the concentration is too high, instead of fibers, helix-shaped micro-ribbons are observed. The molecular weight of the polymer significantly affects the morphology of fibers, due to its effect on the entanglement of polymer chains in solution. When the concentration is fixed, decreasing the molecular weight causes bead, instead of fiber, formation. When increasing the molecular weight, the number of beads and droplets is decreased and smooth fibers are obtained. Considering that each of the solution parameters has an effect on each other, molecular weight reflects the entanglement of the polymer chains, thus it affects viscosity and variance in viscosity can cause different surface tension, which plays an important role in bead formation. The solution viscosity, which can be adjusted by the polymer concentration, is one of the key factors in terms of fiber morphology. Low viscosity prohibits continuous and smooth fiber production, whilst at a high viscosity, longer stress relaxation time occurs, which causes hard ejection of the jets from the polymer solution. At optimal viscosity, uniform in diameter fibers are produced.

Surface tension determines the boundaries of the electrospinning process. Higher surface tension causes instability of the jets and yields sprayed droplets. The solvent and the polymer used as well as the addition of ionizable salts determine the solution conductivity. It has been shown that an increased conductivity causes a decrease in the diameter of the electrospun fibers, whilst low conductivity produces fibers with beads. Natural polymers are polyelectrolytic in nature and the ions present increase the charge carrying capacity of the jet. Further, the addition of ionic salts, such as KH_2_PO_4_, NaH_2_PO_4_ and NaCl, affect fiber morphology and diameter and allow production of bead-less fibers with relatively smaller diameters (Bhardwaj and Kundu, 2010; Li and Wang, 2013).

### Process Parameters

The process parameters (e.g., voltage, flow rate, distance between needle and collector, collector's features) are also playing a crucial role in the production of reproducible fibers. Obviously, the voltage is crucial in the electrospinning process. When the applied voltage overcomes the threshold voltage, fibrous scaffolds can be produced. When the other parameters are fixed, increasing voltage can cause the formation of beads and droplets. With respect to the influence of voltage on fiber diameter, two contradictory theories exist. High voltages are associated with more polymer ejections and thus larger in diameter fibers. On the other hand, increasing the applied voltage results in smaller in diameter fibers due to the electrostatic repulsive force on the fluid jet. Greater Columbic forces and stronger electric field, arising from high voltages, induce increased stretching of the solution, which results in reduction in the fiber diameter and rapid evaporation of the solvent. The flow rate influences the jet velocity and the material transfer rate. High flow rates produce large in diameter fibers, whilst lower flow rates are more desirable for reproducible fiber production, as they provide sufficient drying time to the jet to reach the collector (Subbiah et al., 2005; Pham et al., 2006; Bhardwaj and Kundu, 2010). The morphology of the fibers is also affected by the distance between the tip of the needle and the collector. Too long or too short distances cause beaded morphology; therefore, an optimal distance should be identified to allow the fiber to dry before reaching the collector (Ki et al., 2005; Hassiba et al., 2016). Having said that, a study has argued that of the other parameters are optimal, the distance has no crucial effect on fiber size and morphology (Pham et al., 2006). The collector acts as a conductive substrate, where the charged fibers are collected. Its conductivity affects the arrangement of the fibers, because of its influence on the charge of the deposited fibers; a low in conductivity collector causes the deposited fibers to detain some of their charges and this causes a repelling effect to the incoming fibers. Customarily, flat aluminum collectors are used, but they are often associated with detachment issues that affect morphology and mechanical properties (Stanger et al., 2009; Pillay et al., 2013). Alternative collector conformations include porous metals of variable porosity and pore shape (Fuller et al., 2016, 2019), wire mesh (Wang et al., 2005), pin (Sundaray et al., 2004), grids (Li et al., 2004), liquid bath (Ki et al., 2007), rotating rods or wheels and parallel or gridded bars for anisotropic fiber production (Xu et al., 2004). It is also worth noting that fibers have also been produced using a non-conductive collector and an AC high voltage electrospinning, instead of a normal DC high voltage (Kessick et al., 2004).

### Ambient Parameters

Ambient parameters (e.g., humidity, temperature) also have an impact on the morphology of the fibers. Relative humidity can make the fibers thicker or thinner based on chemical nature of the polymer. In general, high humidity prohibits solvent evaporation and results in beaded or flat mats, as opposed to fibrous mats. High temperatures, due to reduction in surface tension and viscosity, yield small in diameter fibers (De Vrieze et al., 2009).

## Electrospun Polymers in Cartilage Engineering

### Overview of Polymers Used in Cartilage Engineering

Up to now, numerous natural, synthetic and composite polymers have been electrospun and assessed for cartilage engineering; the critical issues for all of them are their compositional, structural, mechanical, degradation, and biocompatibility properties. The degradation products of natural polymers can be smoothly eliminated from the body and that is why they have been used extensively in cartilage repair and regeneration (Table 1). However, their degradation by the harsh solvents used in the electrospinning process (Yang et al., 2008; Zeugolis et al., 2008) requires heavy cross-linking to stabilize them, which frequently associated with cytotoxicity *in vitro* and foreign body response *in vivo* (Delgado et al., 2015), their fast degradation rate for a tissue that has slow recovery time and their potential immune responses and microbial/viral contaminants have restricted the use of natural polymers in the fabrication of electrospun scaffolds for cartilage engineering (Schmidt and Baier, 2000; Lavik and Langer, 2004). Synthetic polymers are in general stronger than natural polymers, can withheld the electrospinning process without any noticeable losses and offer controllable biodegradability (Cheung et al., 2007; Li et al., 2007; Zhang et al., 2009); for these reasons, synthetic (Table 2) and composites (Table 3) polymers are extensively used in cartilage engineering.

**Table 1:** Electrospun natural polymers used in cartilage engineering.

**Polymer**	**Solvent**	**Fiber diameter**	**Fiber orientation**	**Cell type**	***in vivo***	**Reference**
Collagen II	Hexafluoroisopropanol	110–1,750 nm	Random	Human chondrocytes	–	Matthews et al., 2003
Collagen II	Hexafluoroisopropanol	70–2,740 nm	Random	Immortalized human chondrocytes	–	Shields et al., 2004
Chitosan	Proprietary composition	3,000 nm	Aligned	Canine chondrocytes	–	Subramanian et al., 2005
Chitosan	Hexafluoroisopropanol/Methylene chloride	20–300 nm	Random	Bovine chondrocytes	–	Shim et al., 2009
Gelatin	Trifluoroethanol/Glacial acetic acid	100–1,000 nm	Random	Calf chondrocytes	–	Skotak et al., 2010
Keratin	Sodium carbonate–bicarbonate buffer/Sodium dodecyl sulfate	4,800 nm	Random	Human ADSCs	–	Xu et al., 2014

**Table 2:** Electrospun synthetic polymers used in cartilage engineering.

**Polymer**	**Solvent**	**Fiber diameter**	**Fiber orientation**	**Cell type**	***in vivo***	**Reference**
PCL	Tetrahydrofuran /Dimethylformamide	700 nm	Random	Fetal bovine chondrocytes	–	Li et al., 2003
PCL	Chloroform /Dimethylformamide	400–1,400 nm	Random	Human BMSCs	–	Alves da Silva et al., 2010
PCL	Methylene chloride/Dimethylformamide	500–3,000 nm	Aligned	Human BMSCs	–	Wise et al., 2009
PCL	Dimethylformamide/Tetrahydrofuran	500–900 nm	Random	Human BMSCs	–	Li et al., 2005b
PCL	Dimethylformamide/Tetrahydrofuran	300–1,500 nm	Random	Swine chondrocytes Human BMSCs	Swine model	Li et al., 2006a, 2009
PCL	Chloroform/Dimethylformamide	175–875 nm	Random	Human chondrocytes Human Wharton's jelly stem cells	–	Guimarães et al., 2010; Alves da Silva et al., 2017
PCL	Hexafluoroisopropanol	900–4,600 nm	Aligned and random	Bovine Chondrocytes	–	McCullen et al., 2012
PCL	Hexafluoroisopropanol	1,570 ± 500 nm (aligned) 2,340 ± 740 nm (random)	Aligned and random	Rat BMSCs	–	Munir et al., 2019
PCL PCL-Polyurethane	Tetrafluoroethylene/N, N-dimethylacetamide	200–1,600 nm	Random	Human BMSCs	–	Kuo et al., 2014
PLGA	Dimethylformamide/Tetrahydrofuran	400–700 nm	Random	Porcine chondrocytes	–	Shin et al., 2006
PGA PDLLA PLLA PLGA PCL	Hexafluoroisopropanol Tetrahydrofuran/Dimethylformamide Chloroform/Dimethylformamide Tetrahydrofuran/Dimethylformamide	300–1,500 nm	Random	Human BMSCs Bovine chondrocytes	–	Li et al., 2006a
Poly(p-dioxanone) PLGA	Hexafluoroisopropanol	1,220–1,870 nm	Aligned and random	Human BMSCs	–	Rowland et al., 2016
Co-poly(ether)esterurethane Polyetherimide Poly(p-dioxanone)	Hexafluoroisopropanol Dimethylacetamide	2,000–3,500 nm	Aligned and random	Porcine chondrocytes	–	Schneider et al., 2012
PLLA	Chloroform/Dimethylformamide	500–15,000 nm	Random	Bovine chondrocytes	–	Li et al., 2006b
PLLA	Chloroform/Dimethylformamide	300–1,500 nm	Random	Human BMSCs	–	Li et al., 2006a
PLLA	Hexafluoroisopropanol Chloroform Dichloromethane	290–9,000 nm	Random	Human BMSCs	–	Janjanin et al., 2008; Shanmugasundaram et al., 2011
PLA	Dichloromethane/Dimethylacetamide	700–3,840 nm	Aligned and random	Human vascular and avascular meniscus cells	–	Baek et al., 2015
PLG	Dichloromethane	3,000–14,000 nm	Random	–	Rabbit model	Toyokawa et al., 2010

**Table 3:** Electrospun composite polymers used in cartilage engineering.

**Polymers**	**Solvent**	**Fiber diameter**	**Fiber orientation**	**Cell type**	***in vivo***	**Reference**
Hydroxy apatite/PLGA/Collagen I	Hexafluoroisopropanol	421 ± 208 nm	Random	Human stem cells (tissue was not specified)	–	Mouthuy et al., 2010, 2016
PCL/Fibrin	Chloroform/Methanol	250–8,800 nm	Random	Human umbilical cord stem cells	–	Levorson et al., 2013
PCL/Cartilage derived matrix	Hexafluoroisopropanol	560–580 nm	Random	Human ADSCs	–	Garrigues et al., 2014
PLLA/Multi walled carbon nano tubes	Dichloromethane/Dimethylformamide	1,332–3,390 nm	Random	Human BMSCs	–	Holmes et al., 2013
PLGA/Hydroxy apatite/Zein	Hexafluoroisopropanol	200–500 nm	Random	Human umbilical cord stem cells	Rabbit model	Lin et al., 2015
PLLA/Polyethylene glycol/Polyhedral oligomeric silsesquioxane	Chloroform/Dimethylformamide	483–884 nm	Random	Human BMSCs	–	Gomez-Sanchez et al., 2014
Collagen type I/PLCL	Hexafluoroisopropanol	237 ± 65 nm	Random	Rabbit chondrocytes	Mice model	He et al., 2013
PLA/PCL	Chloroform/Dimethylformamide	400–500 nm	Random	Human chondrocytes	–	Thorvaldsson et al., 2008
PLLA/Silk fibroin	Trifluoroacetic acid/Hexafluoroisopropanol	770 ± 160 nm	Random	Rabbit chondrocytes	–	Li et al., 2016
PLDLA nano-fibers/PLDLA micro-fibers	Chloroform/Dimethylformamide	418–728 nm	Aligned and random	Bovine chondrocytes	–	Wimpenny et al., 2012
Gelatin/PCL	Acetic acid/Tetrafluoroethylene	434 ± 130 nm	Random	Coculture of rabbit bone marrow stromal cells and rabbit chondrocytes (75:25)	Mice model	He et al., 2015
PVA/PCL	Chloroform/Dimethylformamide	300–800 nm	Random	Rabbit BMSCs	Rabbit model	Shafiee et al., 2011
PLLA/PCL	Chloroform	100–1,900 nm	Aligned and random	Human nasal septum derived progenitors	–	Shafiee et al., 2014
Poly(3-hydroxybutyrate-co-3-hydroxyvalerate)	Tetrafluoroethylene	600 nm	Random	Rabbit chondrocytes	–	Kwon et al., 2007
Gelatin/PCL	Dichloromethane/Dimethylbenzene/Span 20/Formic Acid/Ethyl ester	305 ± 72 nm	Random	Mouse iPSCs	Rabbit model	Liu et al., 2014a
PDLA/PLLA PDLA/PCL	Dichloromethane/Dimethylformamide Tetrahydrofuran/Dimethylformamide Dichloromethane/Dimethylformamide	503–1,000 nm	Random	Canine chondrocytes	–	Wright et al., 2014
PDLLA/Bioglass®	Dimethyl carbonate	100–200 nm	Random	Mouse chondrocyte cell line	–	Yunos et al., 2013
Poly(3-hydroxybutyrate)/Poly(3-hydroxyoctanoate)	Chloroform	336–744 nm	Random	Human chondrocytes	–	Ching et al., 2016
PLA/Carbon nanotubes/Gelatin	Dichloromethane/Dimethylformamide Acetic acid	112–289 nm	Random	Human chondrocytes	–	Markowski et al., 2015
PLCL/Collagen type I	Hexafluoroisopropanol	20,000 ± 10,000 nm	Aligned and honeycomb	Rabbit BMSCs	–	Zheng et al., 2016
Poly(vinyl alcohol) methacrylate/Poly(vinyl alcohol) methacrylate-Chondroitin sulfate methacrylate	Ultra-pure water	410–500 nm	Random	Goat BMSCs	Rat model	Coburn et al., 2012
Gelatin/PLLA	Methylene chloride/Dimethylformamide	222 ± 14 nm	Random	Rabbit chondrocytes	Rabbit model	Chen and Su, 2011
Hyaluronic acid/Collagen I	Sodium hydroxide/Dimethylformamide	226–357 nm	Random	Bovine chondrocytes	–	Kim et al., 2008
PCL/Collagen I/Hyaluronic acid/Tricalcium phosphate	Chloroform	6,480 ± 1,640 nm	Aligned	Human BMSCs	Rabbit model	Liu et al., 2014b
PLGA/3,4,6-O-Bu3GlcNAc	Dichloromethane	20,000–2,000 nm	Random	Human chondrocytes	Rat model	Kim et al., 2016
PLA/Gelatin PLA/Gelatin/Hyaluronic acid	Hexafluoroisopropanol	Not specified	Random	Rat chondrocytes	Rabbit model	Chen et al., 2016
Poly(ethylene oxide-terephthalate)/Poly(butylene terephtalate)	Chloroform/Hexafluoroisopropanol	10,000 ± 2,800 nm	Random	Bovine chondrocytes	–	Moroni et al., 2008
PCL/PLA	Chloroform/Ethanol	1,430–3,160 nm	Random	–	Rabbit model	Islas-Arteaga et al., 2018
PLLA/PEG	Chloroform/Dimethylformamide	1,000 nm	Random	Chondrocytes/Stem cells (Species and tissue were not specified)	–	Mirzaei et al., 2017
PCL/PEO	Tetrahydrofuran/Dimethylformamide/Ethanol	471 ± 133 nm	Aligned	Rabbit synovial stem cells	Rabbit model	Shimomura et al., 2019
Poly(hydroxybutyrate)/Chitosan ± Al_2_O_3_	Trifluoroacetic acid	300–550 nm	Random	Rabbit chondrocytes	–	Sadeghi et al., 2016; Toloue et al., 2019
PLA/Gelatin/Resveratrol	Hexafluoroisopropanol	200–2,200 nm	Random	–	SD rat model	Yu et al., 2018
Cellulose/Silk	Trifluoroacetic acid/glacial acetic acid	68 ± 17 nm	Random	Human BMSCs	–	Begum et al., 2018
PCL/Phytochemicals	Ethyl acetate	316 ± 7 nm	Random	Human meniscus cells	–	Venugopal et al., 2019
PCL/Graphene oxide/Collagen microporous construct	Dichloromethane/Dimethylformamide	1,200–2,000 nm	Random	–	–	Girão et al., 2018
Sodium cellulose sulfate/Gelatin	Deionized water/Ethanol	1,700–3,700 nm	Random	Human BMSC pellets	–	Huang et al., 2018
Poly (3-hydroxybutyrate)/Chitosan/β-Tricalcium phosphate	Trifluoroacetic acid	400–1,200 nm	Random	Rabbit chondrocytes	–	Keikhaei et al., 2019
PLLA/Polydopamine/Chondroitin sulfate	Dichloromethane	3,000–7,000 nm	Aligned	Rabbit chondrocytes/Rabbit BMSCs	Rabbit model	Ren et al., 2019
PCL/PLGA	Chloroform/Dimethylformamide	400–1,200 nm	Random	Human BMSCs	–	Zamanlui et al., 2018
Gelatin/Chondroitin sulfate	Tetrafluoroethylene/Water	189–230 nm	Random	Human BMSCs	–	Honarpardaz et al., 2019
PCL/Polytetrahydrofuran urethane/Collagen I	Hexafluoroisopropanol	444 ± 67 nm	Random	Rat femoral marrow stem cells	SD rat model	Jiang et al., 2018
Gelatin/PLGA	Hexafluoroisopropanol	~1,000 nm	Random	Primary rabbit chondrocytes	Mice model	Chen et al., 2019

Poly(α-hydroxy esters) [e.g., poly(glycolic acid) (PGA), poly(lactic acid) (PLA), poly(ε-caprolactone) (PCL) and their copolymers] are used extensively for tissue engineering applications, as they are well-characterized and FDA approved for clinical use. The simplest linear aliphatic polyester is PGA. It is considered as a promising biomaterial due to the natural absorption of its degradation products; however, its rapid degradation rate makes it an inappropriate candidate for cartilage engineering. PLA is more hydrophobic than PGA with an addition of a methyl group; however, it is readily soluble in commonly used organic solvents. Based on the position of the methyl group, it has three isomers, which are poly(L-lactic acid) (PLLA), poly(D,L-lactic acid) (PDLLA), and poly(D-lactic acid) (PDLA). Compared to PGA, PLA degrades slowly (from 1 to over 2 years) because of the hydrophobic characteristics. However, the tensile strength and modulus of elasticity of PLA is lower than PGA. Although the use of PLA and PGA is limited for hard tissue regeneration, such as cartilage tissue, due to their relatively weak mechanical properties (Cheung et al., 2007), one study showed that bidirectionally aligned and layered PLA electrospun mats loaded with human meniscus cells in an ECM hydrogel displayed ~5-fold higher tensile modulus to the randomly aligned scaffolds; they had comparable tensile modulus to the human meniscus in the circumferential direction and they maintained physiological meniscus cells gene expression for COLA1A1, SOX9, and COMP (Baek et al., 2015).

In general, electrospun copolymers of poly(α-hydroxy esters) with tailored properties can be readily obtained and are extensively used in tissue engineering and regenerative medicine. When six commercially available poly(α-hydroxy esters) were incubated in physiological solutions, the PGA and PLGA50:50 scaffolds showed superior mechanical properties than the PLLA and PCL scaffolds; the PLLA and PCL scaffolds sustained their robust scaffold structure; and the PGA, PDLLA, PLGA50:50, and PLGA85:15 scaffolds exhibited a severe structural destruction due to polymer degradation. In terms of cell proliferation, PLLA scaffolds promoted the highest rate of proliferation between all polymers when seeded with chondrocytes and human BMSCs (Li et al., 2006a). A study that compared different PLGA ratios (75:25, 50:50) and a blend of 75:25 and 50:50 PLGA showed that the tensile modulus of the 75:25 and 50:50 PLGA scaffolds were similar to human skin and slightly lower than human cartilage, respectively (Shin et al., 2006). Due to its relatively cheap cost, high stability in ambient conditions, long degradation rate and the long regeneration time of cartilage tissue, PCL is favored in cartilage repair and regeneration, with numerous studies having demonstrated that electrospun PCL scaffolds promote cartilage cell proliferation, cartilage ECM synthesis and deposition and chondrogenic differentiation of various stem cell populations. Further, PCL nanofibrous scaffolds have shown higher chondrogenic differentiation, as judged by sGAG synthesis, of BMSCs than cell pellet cultures in TGF-β1 serum free media (Li et al., 2005b).

### The Influence of Architectural Features on Cell Response

Over the years, numerous studies gave assessed the influence of architectural features (e.g., fiber orientation, fiber diameter, scaffold porosity) on cell fate. It has been shown that both aligned and random PLLA/PCL (Shafiee et al., 2014) and PCL/PLGA (Zamanlui et al., 2018) scaffolds support nasal septum-derived progenitor and human BMSCs, respectively, adhesion, proliferation and chondrogenesis. However, their proliferation was higher on the random scaffolds, whilst their differentiation was higher on the aligned scaffolds, rendering such conformation suitable for the superficial zone of the articular cartilage that exhibits an aligned orientation. Although both aligned nano- and micro- fibrous electrospun PCL scaffolds sustained growth of human BMSCs, the nano-fibrous scaffolds showed the highest chondrogenic activity, as judged by produced sGAG and collagen type II mRNA expression, suggesting that this combination may be suitable form for the superficial zone, which normally shows the highest level of collagen type II than the any other zone (Wise et al., 2009). Similar results were obtained with nano-fibrous, as opposed to micro-fibrous or smooth (film) PLLA (Li et al., 2006b) or PLDLA (Wimpenny et al., 2012) scaffolds; the nano-fibrous architecture maintained chondrocyte-like morphology and enhanced cartilage-specific mRNA expression and ECM synthesis. One should however note that not only the fiber size, but also the pore size has an important role in chondrogenesis. For example, micro-size PLLA fibers of 5 and 9 μm in diameter and with pore sizes of 27 and 29 μm respectively were more chondrogenic (e.g., aggrecan, chondroadherin, sox9, collagen type II) than nano-size PLLA fibers of 300 nm and 600 nm to 1,400 nm in diameter and with pore sizes of 2 and 3 μm respectively (Shanmugasundaram et al., 2011).

### Electrospinning and Bioreactors

Considering that cells *in vivo* are subjected to numerous tissue-specific cues, modern molecular delivery (Pugliese et al., 2018) and tissue engineering (Calejo et al., 2019) employ multifactorial approaches to recapitulate the *in vivo* niche *in vitro*. To this end, electrospun fibers have joined forces with other *in vitro* microenvironment modulators to either maintain native chondrocyte phenotype or to direct stem cells toward chondrogenic lineage, especially now that it is clear that a stable chondrocyte phenotype is still elusive (Graceffa et al., 2018, 2019). For example, dynamic culture systems combined with electrospun scaffolds have shown beneficial effects in cartilage engineering (Martin et al., 2007; Janjanin et al., 2008; Khorshidi et al., 2016). A flow perfusion bioreactor, promoted chondrogenic differentiation of human BMSCs, as judged by increased expression of cartilage-associated genes (e.g., aggrecan, collagen type II, SOX9) and enhanced cell proliferation and ECM synthesis. However, there was no significant difference between bioreactor culture and static control culture, suggesting that the media fluid flow and the orientation of the electrospun meshes can also have an impact (Alves da Silva et al., 2010). Using a custom mold, PLLA electrospun scaffolds seeded with BMSCs and media supplemented with TGF-β1/IGF-1, after 42 days in a bioreactor system, the produced construct exhibited the highest (in comparison to TGF-β1 alone culture) Young's modulus values and collagen type II and aggrecan expression; a significant time-dependent increase in sGAG and hydroxyproline content was also reported (Janjanin et al., 2008).

### Improving Cell Infiltration and Nutrient/Waste Transport

Highly dense/small porosity electrospun scaffolds often cause low cell infiltration and limited nutrient access to the deeper sides of the cartilage tissue (Nam et al., 2007; Skotak et al., 2011; Coburn et al., 2012). To enhance cellular infiltration and nutrient/excrete transport, various ingenious engineering approaches have been assessed over the years, including combination of nano-micro fibrous scaffolds (Kim et al., 2008; Thorvaldsson et al., 2008; Levorson et al., 2013), salt leaching techniques (Wright et al., 2014), controlled fiber density (Coburn et al., 2012), electrospinning in liquids (Thorvaldsson et al., 2008), and sacrificial fibers (Baker et al., 2008; Whited et al., 2011), with remarkable results. For example, an electrospun scaffold comprised of PCL microfibers and fibrin nanofibers resulted in higher human umbilical cord blood MSCs infiltration and GAG synthesis than PCL microfibres and PCL micro- and nano-fibers (Levorson et al., 2013). Electrospinning of PVA/methacrylate/chondroitin sulfate in ethanol bath enhanced goat BMSCs infiltration, proliferation and chondrogenesis *in vitro* and cartilage regeneration *in vivo*, even without cells or any other exogenous factor (Thorvaldsson et al., 2008). Salt leaching of chitosan hydrogels reinforced with either PDLA/PLLA or PDLA/PCL has been shown to increase porosity; however, the PDLA/PLLA-based scaffolds provided a favorable elastic modulus for articular cartilage, whilst the PDLA/PCL-based scaffolds exhibited better biological response (Slivka et al., 2001; Wright et al., 2014).

### From Two-Dimensional to Three-Dimensional Constructs

To more closely imitate the native three-dimensional cartilage architecture, multi-layer horizontally, randomly, and vertically aligned fibers PCL fibers in a graphene-oxide-collagen microporous network have been developed (Girão et al., 2018). To imitate the three-dimensional cartilage architecture and composition, PCL/cartilage-derived matrix electrospun fibers were produced in single- and multi- layered conformations; the resultant multi-layered scaffolds enhanced chondrogenesis of human ADSCs, as judged by increased sGAG synthesis and increased gene expression of collagen type X, but had lower elastic modulus to PCL-alone scaffolds (Garrigues et al., 2014). Despite these significant advancements, scalability of such constructs is of concern. For this reason, electrospinning has been combined with other fabrication technologies for the development of three-dimensional constructs that closely imitate native cartilage architectural features. For example, electrospinning with rapid prototyping resulted in scaffolds with acceptable mechanical properties that supported bovine chondrocyte growth and cartilage-ECM synthesis for 4 weeks *in vitro* (Moroni et al., 2008). Electrospinning combined with freeze-drying has been shown to yield scaffolds that supported rabbit BMSC growth *in vitro* (Zheng et al., 2016) and to successfully regenerate osteochondral defects in a rabbit model (Liu et al., 2014b). More complex scaffolds have also been prepared and demonstrated efficacy in a mice model using electrospinning, three-dimensional printing and freeze drying (Chen et al., 2019).

### Preclinical Data

It is worth noting that all small animal *in vivo* data have shown promising results. For example, in nude mice, layer-by-layer sandwich constructs of collagen/PLCL seeded with rabbit auricular chondrocytes reached 83% Young's modulus of native auricular cartilage after 12 weeks of implantation (He et al., 2013). PVA with chondroitin sulfate electrospun fibers in a rat osteochondral defect model resulted in enhanced chondrogenesis, compared to the empty control group (Coburn et al., 2012). Resveratrol-PLA-gelatin scaffolds resulted in faster healing than PLA-gelatin scaffolds in a rat articular cartilage defect model 12 weeks post-implantation (Yu et al., 2018). In a rabbit cartilage defect model, aligned PLLA-polydopamine-chondroitin sulfate fibers facilitated the filling of defects and the regeneration of hyaline cartilage-like tissue (Ren et al., 2019). In rabbit meniscal defects, aligned PCL fibers (produced with sacrificial PEO fibers) combined with a tissue engineered construct derived from synovial mesenchymal stem cells significantly contributed to the prevention of meniscal extrusion, exerted a chondroprotective effect and meniscal defects were repaired with a fibrocartilaginous tissue (Shimomura et al., 2019). In the only large animal model *in vivo* work (7 mm full thickness cartilage defect swine model), PCL fibers loaded with human BMSCs showed the most complete repair, generated hyaline cartilage-like tissue and had the highest equilibrium compressive stress of 1.5 MPa in the regenerated cartilage after 6 months of implantation, in comparison to PCL scaffolds alone and PCL/allogenic chondrocytes constructs (Li et al., 2009). Despite these profound preclinical data, no clinical studies are available to-date in cartilage engineering.

### Critical Analysis and Outlook

Electrospinning has been adopted in tissue engineering and regenerative medicine since the 1990's. Since then, a substantial amount of work has been conducted, as evidenced by the wealth of scientific publications available (e.g., 8,103 papers in PubMed; term searched “electrospinning” in all fields). In cartilage space, the electrospinning technology is still at its infancy, which can be substantiated by the low number of scientific publications available (e.g., 155 papers in PubMed; terms searched “electrospinning” and “cartilage” in all fields). Nonetheless, significant strides (e.g., development of three-dimensional tissue equivalents that, to a certain extent, replicate the complex cartilage architecture and composition and have resulted in promising *in vivo* data in small animal preclinical models) have been achieved. However, it is also apparent that large animal experimentation and clinical translation are lagging behind for cartilage and also other clinical indications (e.g., only 5 clinical studies appear at clinicaltrials.gov, term searched “electrospinning” in all studies). This limited technology transfer from benchtop to large animal models and to clinical setting may be attributed to scalability and infrastructure costs required to produce reproducible fibers (e.g., controlled temperature/humidity chambers, automated systems, variable collectors, multi-syringe systems). Considering though that electrospun scaffolds have started becoming commercially and clinically available (Ryan et al., 2015), we believe that in the years to come they will also be assessed in cartilage engineering.

We also believe that in the years to come electrospun scaffolds together with other *in vitro* microenvironment modulators will play a crucial role in the development of functional cell therapies for cartilage engineering. For example, the positive impact of bioreactors in musculoskeletal tissue engineering has been well-established (Peroglio et al., 2018) and electrospun scaffolds coupled with bioreactors have shown promise to-date, even for complex structures, such as the cartilage-bone interface (Baumgartner et al., 2019). Further, considering that extracellular matrix is key modulator of cell fate through provision of biophysical, biochemical, and biological signals (Guilak et al., 2009; Watt and Huck, 2013; Kumar et al., 2017; Muncie and Weaver, 2018; Smith et al., 2018; Novoseletskaya et al., 2019), strategies that enhance and accelerate native extracellular matrix synthesis [e.g., hypoxia (Taheem et al., 2019)] and deposition [e.g., macromolecular crowding (Graceffa and Zeugolis, 2019)] coupled with electrospinning are likely to lead to more biomimetic three-dimensional cartilage equivalents. It is also worth noting, that although the cell-sheet/scaffold-free technology has shown promise in human cartilage engineering (Sato et al., 2019), only thin layers of tissue can be developed, which imposes the need of either multi-layered approaches that are often associated with delamination and cell death in the middle layers due to poor nutrient/waste transport (Sekine et al., 2011) or multiple surgeries (Shimizu et al., 2006; Komae et al., 2017). Considering that advances in engineering are now allowing the development of porous electrospun scaffolds (Ameer et al., 2019), we believe that temperature-responsive electrospun scaffolds will play a key role in the development of scaffold-free three-dimensional tissue-like surrogates in the years to come.

## Conclusions

Electrospinning can produce nano- to micro-range fibrous constructs that closely imitate the architecture of native tissues. Further, has the capacity to deliver cells and therapeutic molecules at the side of injury. Advancements in fabrication methods have addressed scalability issues and have allowed the development of porous structures than enable cell infiltration and growth for prolonged periods of times. Despite all these advantages, electrospun scaffolds have yet to be assessed comprehensively in preclinical models and clinical setting, which has compromised wide acceptance of this pioneering technology in biomedicine.

## Author Contributions

All authors listed have made a substantial, direct and intellectual contribution to the work, and approved it for publication.

### Conflict of Interest

The authors declare that the research was conducted in the absence of any commercial or financial relationships that could be construed as a potential conflict of interest.
